# Assessing Criminal Responsibility in a Young Offender With Autism Spectrum Disorder: A Forensic Case Study

**DOI:** 10.1192/bjo.2026.11785

**Published:** 2026-06-30

**Authors:** Fawaz Gali, Reem Mohammad Saleem, Muhanad Elnoor, Pearl Rex, Mohammed Ibrahim

**Affiliations:** 1 Al Ain Hospital, Al Ain, UAE; 2 Al Amal Hospital, Dubai, UAE; 3 Betsi Cadwaladr University Health Board, Wrexham, United Kingdom

## Abstract

**Aims:**

Autism Spectrum Disorder (ASD) is associated with social communication deficits, atypical social cognition, and behavioural regulation difficulties, which can complicate forensic evaluations of criminal responsibility. This case study aims to illustrate the assessment process and clinical reasoning used to determine criminal responsibility in a young offender with ASD and below-average intellectual functioning.

**Methods:**

A 17-year-old male was referred for forensic psychiatric assessment following an alleged vehicle theft and reckless driving. A multidisciplinary evaluation was undertaken including psychiatric assessment (developmental, behavioural, substance use history and mental state examination) and clinical psychological testing. ASD assessment used ADI-R and ADOS; cognitive functioning was assessed using the Stanford-Binet Intelligence Scales (Fifth Edition).

**Results:**

Assessment findings supported a diagnosis of ASD with moderate symptom severity and significant social communication deficits. Cognitive testing showed below-average intellectual functioning (Full-Scale IQ 82) with relative strengths in visual-spatial processing and weaknesses in quantitative reasoning and knowledge domains. Clinically, the patient demonstrated limited insight into broader social consequences and reduced emotional reciprocity, without evidence of psychosis. Despite neurodevelopmental vulnerabilities and contextual risk factors (including alcohol use), the forensic conclusion was that the capacity to distinguish right from wrong at the time of the alleged offence was preserved, and criminal responsibility was not negated. The case highlights how ASD-related impairments may affect judgement and impulsivity while still allowing intact understanding of wrongfulness, underscoring the need for structured, ASD-informed forensic formulation.

**Conclusion:**

Forensic assessments of adolescents with ASD should integrate developmental history, structured ASD measures, cognitive profiling, and careful analysis of moral/legal understanding at the material time. This case supports a nuanced approach that balances legal accountability with neurodevelopmental formulation and recommends tailored interventions (e.g., behavioural therapy, social skills work and family psychoeducation) to reduce future risk.

